# Pain Perception and Dietary Impact in Fixed Orthodontic Appliances vs. Clear Aligners: An Observational Study

**DOI:** 10.3390/jcm14145060

**Published:** 2025-07-17

**Authors:** Bianca Maria Negruțiu, Cristina Paula Costea, Alexandru Nicolae Pîrvan, Diana-Ioana Gavra, Claudia Judea Pusta, Ligia Luminița Vaida, Abel Emanuel Moca, Raluca Iurcov, Claudia Elena Staniș

**Affiliations:** 1 Department of Dental Medicine, Faculty of Medicine and Pharmacy, University of Oradea, 10 Piața 1 Decembrie Street, 410073 Oradea, Romania; bnegrutiu@uoradea.ro (B.M.N.); cristinacostea@uoradea.ro (C.P.C.); ligia_vaida@uoradea.ro (L.L.V.); 2 Department of Surgical Disciplines, Faculty of Medicine and Pharmacy, University of Oradea, 10 Piața 1 Decembrie Street, 410073 Oradea, Romania; alexandrupirvan88@gmail.com; 3 Department of Preclinical Disciplines, Faculty of Medicine and Pharmacy, University of Oradea, 10 Piața 1 Decembrie Street, 410073 Oradea, Romania; diannagavra@yahoo.com; 4 Department of Morphological Disciplines, Faculty of Medicine and Pharmacy, University of Oradea, 10 Piața 1 Decembrie Street, 410073 Oradea, Romania; claupustaml@yahoo.com; 5 Department of Medical Disciplines, Faculty of Medicine and Pharmacy, University of Oradea, 10 Piața 1 Decembrie Street, 410073 Oradea, Romania; stanis.claudia@yahoo.com

**Keywords:** humans, malocclusion, pain, analgesics, surveys and questionnaires, weight loss, eating habits, orthodontic appliances

## Abstract

**Background and Objectives:** Orthodontic treatment, whether fixed or removable, offers several benefits, including improved aesthetics, enhanced oral function, and increased self-confidence. However, it may also cause discomfort and pain, particularly following adjustment visits. This study aimed to assess pain characteristics (latency and continuity), food impairment, weight loss, and analgesic use in relation to treatment duration and appliance type. **Methods:** This observational study included 160 orthodontic patients who completed a structured questionnaire comprising 13 single-choice items. The questionnaire assessed age, gender, residential environment, educational status, type and duration of orthodontic treatment, pain characteristics (duration, latency, continuity), food impairment, and analgesic use. Inclusion criteria specified patients with moderate anterior crowding undergoing fixed orthodontic treatment or treatment with clear aligners on both arches, for at least one month. All fixed appliance cases involved 0.022-inch-slot Roth prescription brackets. **Results:** Patients undergoing fixed orthodontic treatment reported a higher frequency of pain (91.4%), greater need for analgesics (95.2%), and more food impairment compared to those with clear aligners. Patients treated for less than 6 months more frequently reported pain lasting 1 week (57.1%), while those treated for 1–2 years more commonly reported pain lasting several days (43.8%). **Conclusions:** Fixed orthodontic appliances are associated with greater discomfort, longer pain latency, more frequent analgesic use, and higher dietary impact compared to clear aligners. These findings emphasize the importance of personalized patient counseling and proactive pain management to improve compliance, enhance quality of life, and support informed decision-making in orthodontic care.

## 1. Introduction

Orthodontic treatment, whether using fixed appliances or clear aligners, aims to achieve both aesthetic and functional outcomes. However, it can also induce discomfort, depending on the patient’s physical and psychological state [1,2]. Although the exact mechanism through which orthodontic forces elicit discomfort is not fully understood, the current evidence suggests that it involves inflammatory, vascular, cellular, immunological, and neural responses to the orthodontic appliance [3].

The degree of discomfort experienced varies with the type of appliance used. Because clear aligners apply intermittent forces, they allow for tissue reconfiguration between adjustments, generally resulting in reduced pain and discomfort compared to fixed appliances [4]. Furthermore, patients treated with clear aligners report lower levels of anxiety and demonstrate better oral health-related quality of life than those treated with fixed appliances [5]. Alturki et al. (2024) highlighted that individuals undergoing clear aligner treatment perceived significantly less pain within the first 24 h after adjustment [6]. To minimize pain and prevent bracket breakage, orthodontists often advise patients to avoid hard or sticky foods. These dietary restrictions can lead to altered eating habits and potential weight loss during treatment [7].

Few studies have comparatively assessed the impact of fixed orthodontic appliances versus clear aligners on orthodontic pain, weight loss, and analgesic use [5,6]. This study aims to contribute to the existing literature by providing a more comprehensive understanding of pain experiences during orthodontic treatment.

The primary aim of this observational study was to evaluate how the type of orthodontic appliance, fixed versus clear aligners, affects pain perception, particularly in terms of latency and continuity. Secondary objectives included assessing the relationship between treatment duration and pain characteristics, as well as examining associated factors such as dietary changes, weight loss, and the use of analgesics.

The following null hypotheses were tested in this study:

- There is no significant association between the duration of orthodontic treatment and (a) pain latency, (b) continuity of pain, and (c) weight loss.
- There is no significant association between the type of orthodontic treatment and (a) pain latency, (b) dietary impact, and (c) use of analgesics.
- There is no significant association between the duration of fixed orthodontic treatment (less than six months vs. more than one year) and the intensity or duration of pain.

## 2. Materials and Methods

### 2.1. Ethical Considerations

This study was conducted in accordance with the ethical principles outlined in the 1964 Declaration of Helsinki and its subsequent amendments. Ethical approval was granted by the Research Ethics Committee of the University of Oradea (Approval No. 52/10 October 2023). Informed consent was obtained from all participants or their legal guardians prior to enrollment. Before completing the questionnaire, participants were informed that their involvement was entirely voluntary and anonymous, and that no financial or other incentives were offered.

### 2.2. Participants and Data Collection

This investigation was designed as a cross-sectional observational study and is reported in accordance with the STROBE (Strengthening the Reporting of Observational Studies in Epidemiology) guidelines. This study was conducted between October 2023 and June 2024 in two orthodontic settings located in Oradea, Romania: the University Dental Clinic affiliated with the Faculty of Medicine and Pharmacy at the University of Oradea, and a large private orthodontic practice collaborating with the university. A total of 160 orthodontic patients were recruited during routine follow-up visits, all of whom were actively undergoing treatment at the time of participation. The same research team supervised data collection across both sites to ensure methodological consistency. Participants completed a paper-based questionnaire, written in Romanian and designed to be easily understood. Questionnaires were distributed and explained by trained research staff (including academic researchers and postgraduate orthodontic students), who were available to clarify any uncertainties without influencing responses. Patients completed the forms independently in the reception area prior to their clinical appointments.

The questionnaire addressed age, gender, residential setting, educational status, type of orthodontic appliance (fixed or removable), duration of treatment (<6 months, 6–12 months, 1–2 years, >2 years), pain latency (no pain, 5 min, several hours, one day, one week), presence of food impairment, dietary changes, use of analgesics, pain duration (several days, one week), pain continuity (continuous, discontinuous), and self-reported weight loss (Table 1). Pain latency was defined as the self-reported time elapsed between the most recent appliance activation, either wire adjustment (for fixed appliances) or aligner tray change (for clear aligners), and the initial onset of perceived pain. Patients were asked to reflect on their most recent experience of appliance adjustment or tray change.

The questionnaire underwent a validation process to ensure its reliability and relevance to this study’s objectives. Initially, the items were reviewed by six orthodontic specialists for content validity. Minor revisions were made to enhance clarity and clinical relevance. The revised version was pilot-tested on 30 eligible orthodontic patients. Feedback regarding comprehension and ease of completion was collected, leading to further adjustments to improve readability. Internal consistency was assessed using Cronbach’s alpha, which yielded a value of 0.719, indicating good reliability and consistency across items.

Inclusion criteria comprised patients with moderate anterior crowding in both arches who had been undergoing fixed orthodontic treatment or treatment with clear aligners for at least one month. Moderate anterior crowding was assessed based on Little’s Irregularity Index, with values in the range of 4–6 mm [8]. Eligible participants were non-syndromic, without craniofacial deformities, cleft lip or palate, or a history of prior orthodontic treatment. They also had no general health conditions or medication use that could influence pain perception (e.g., rheumatoid arthritis, lupus, arthritis, joint or bone disorders, acute gout).

Exclusion criteria comprised complex orthodontic cases requiring extractions or orthognathic surgery, allergies to orthodontic materials (e.g., nickel), chronic pain conditions, orofacial pain unrelated to orthodontic treatment, active dental caries, or periodontal disease. Participants with systemic health conditions that could affect pain perception, or with gnathological dysfunctions, such as temporomandibular disorders (TMDs) or occlusal abnormalities, were also excluded. Additionally, questionnaires with missing or incomplete responses to key variables were excluded from the final analysis. No imputation methods were applied.

All patients in the fixed appliance group were treated using 0.022-inch-slot Roth prescription brackets on both dental arches. Patients in the clear aligner group were treated with in-house fabricated aligners. The clinical protocol involved aligner changes every two weeks, with each aligner designed to achieve approximately 0.25 mm of tooth movement. Composite attachments were placed following standard biomechanical guidelines, and interproximal reduction (IPR) was performed when necessary, based on a digital setup.

### 2.3. Statistical Analysis

Data analysis was conducted using IBM SPSS Statistics v25 (IBM Corp., Armonk, NY, USA), with visualizations created in Microsoft Excel and Word 2024 (Microsoft Corp., Redmond, WA, USA). Categorical variables were summarized using counts and percentages. Group comparisons were performed using Fisher’s exact test.

To assess the strength and direction of associations identified in contingency tables, univariate binomial logistic regression models were applied. Model outputs included odds ratios (ORs) with 95% confidence intervals (CIs) and corresponding *p*-values. Statistical significance was set at α = 0.05.

Sample size estimation was performed using G*Power 3.1.9.7 (Heinrich Heine University, Düsseldorf, Germany). A power analysis for logistic regression (two-tailed, α = 0.05, power = 0.80, medium effect size, OR = 1.68, df = 1) indicated that a minimum sample of 150 participants was required. The final sample of 160 patients provided sufficient statistical power to detect meaningful associations. Post hoc power analyses were conducted to address concerns regarding the imbalance in group sizes between patients treated with fixed appliances (*n* = 134) and those treated with clear aligners (*n* = 26). Using G*Power version 3.1.9.7 (Heinrich Heine University, Düsseldorf, Germany), statistical power for each of the three primary outcome comparisons was assessed. The calculated power was 1.0 for pain latency (short latency: “no pain” or “5 min” vs. long latency: “≥several hours”), 0.99999 for food impairment (present vs. absent), and >0.999 for analgesic use (present vs. absent). These results confirm that, despite unequal subgroup sizes, the sample was sufficiently powered to detect clinically meaningful differences between the two orthodontic treatment modalities.

Generative AI was used to refine the language of the manuscript. Specifically, OpenAI’s ChatGPT (version GPT-4, OpenAI, San Francisco, CA, USA) was employed to enhance grammar, clarity, and fluency. The authors have reviewed and verified all content to ensure its scientific accuracy and integrity.

## 3. Results

Based on the inclusion and exclusion criteria, the final study sample consisted of 160 orthodontic patients. Regarding age distribution, 26 participants (16.25%) were aged 6–12 years, 82 participants (51.25%) were aged 13–18 years, and 52 participants (32.5%) were older than 18 years. In terms of gender, the majority were female (*n* = 120, 75%), while male participants accounted for 25% (*n* = 40). As for residential environment, most participants resided in urban areas (*n* = 124, 77.5%), whereas 22.5% (*n* = 36) lived in rural areas (Table 2).

Orthodontic pain was commonly reported, with most patients experiencing discomfort for several days (91.3%). The latency of pain onset was most frequently reported as several hours (40%) or one day (28.7%). In most cases, the pain was described as discontinuous (80%). A significant proportion of patients reported lifestyle changes associated with orthodontic treatment, including food impairment (43.8%), dietary changes (47.5%), weight loss (30%), and use of analgesics (52.5%).

The data from Table 3 show the distribution of patients according to age, gender, and pain duration. Most patients (91.8%) reported pain lasting several days, while 8.2% experienced pain for one week. No significant associations were found between pain duration and gender.

Analgesic use was reported by 55.7% of patients, with a significantly higher prevalence among females compared to males (63.5% vs. 46.7%, *p* = 0.021). No significant association was found with residential background in the overall sample (*p* = 0.083) (Table 4).

Table 5 presents the distribution of orthodontic patients according to the type of appliance and related clinical variables. Pain latency differed significantly between treatment groups (*p* < 0.001). Z-tests with Bonferroni correction revealed that patients with fixed orthodontic appliances more frequently reported pain latency of one day or several hours, compared to those reporting no pain or only a 5 min latency (100%/90.6% vs. 71.4%/52.9%). Conversely, patients undergoing treatment with clear aligners were more likely to report no pain or only a 5 min latency, in contrast to those reporting pain onset after several hours or one day (28.6%/47.1% vs. 0%/9.4%). Furthermore, patients with fixed appliances more frequently reported food impairment (91.4% vs. 77.8%, *p* = 0.020) and the use of analgesics (95.2% vs. 71.1%, *p* < 0.001) following appliance adjustments.

Table 6 presents the distribution of orthodontic patients based on treatment duration and related clinical variables. The analysis revealed several statistically significant associations:

- Pain latency differed significantly across treatment durations (*p* < 0.001). Among patients treated for less than 6 months, a significantly higher proportion reported pain lasting 1 week (57.1%) compared to those reporting pain for several days (16.4%). In contrast, among patients treated for 1 to 2 years, the proportion of those reporting pain for several days was significantly higher (43.8%) than those reporting pain lasting one week (0%).
- Pain continuity also showed significant variation by treatment duration (*p* = 0.015). Among patients treated for less than 6 months, discontinuous pain was more frequently reported (23.4%) than continuous pain (6.2%). Conversely, among those treated for more than 2 years, continuous pain was more frequently reported (18.8%) compared to discontinuous pain (4.7%).
- Weight loss was significantly associated with treatment duration (*p* = 0.030). Among patients undergoing treatment for more than 2 years, the proportion of those who did not report weight loss was significantly higher (10.7%) compared to those who experienced weight loss (0%).

**Table 6:** Association between treatment duration and pain latency, pain continuity, and weight loss.

Variable	Category	<6 Months (*n*, %)	6 Months–1 Year (*n*, %)	1 Year–2 Years (*n*, %)	>2 Years (*n*, %)	*p*-Value	Odds Ratio (95% CI)
**Pain** **Duration**	Several days	24 (16.4%)	46 (31.5%)	64 (43.8%)	0 (0.0%)	<0.001 *	N/A
	One week	8 (57.1%)	6 (42.9%)	12 (8.2%)	0 (0.0%)		
**Pain** **Continuity**	Discontinuous	30 (23.4%)	42 (32.8%)	50 (39.1%)	6 (4.7%)	0.015 *	0.067 (0.009–0.504)
	Continuous	2 (6.3%)	10 (31.3%)	14 (43.8%)	6 (18.8%)		
**Weight** **Loss**	Absent	18 (16.1%)	36 (32.1%)	46 (41.1%)	12 (10.7%)	0.030 *	N/A
	Present	14 (29.2%)	16 (33.3%)	18 (37.5%)	0 (0.0%)		

* Fisher’s exact test.

## 4. Discussion

This observational study aimed to compare pain perception, dietary impact, and analgesic use among patients undergoing fixed orthodontic treatment versus those treated with clear aligners. Additionally, it sought to evaluate how treatment duration influences pain latency, pain continuity, and weight loss. The main findings indicate that patients with fixed appliances experienced significantly more frequent and prolonged pain, greater food impairment, and a higher need for analgesics compared to those with clear aligners. Furthermore, shorter treatment duration was associated with increased pain intensity and greater reported weight loss. Nowadays, patients are increasingly involved in choosing their orthodontic appliances, as they are more aware of the importance of oral health and its impact on daily life [9,10]. Consequently, their quality of life during orthodontic treatment is strongly influenced by the degree of pain and discomfort they experience [11].

According to the findings of this study, patients treated with fixed appliances reported significantly longer pain latency compared to those with clear aligners. The majority of fixed appliance users experienced pain onset ranging from several hours to 1 day, while those with clear aligners reported pain latency of only 5 min to several hours. Additionally, patients undergoing orthodontic treatment for less than 6 months experienced high levels of pain for up to a week, whereas those treated for 1–2 years reported such levels for only a few days [12]. These results suggest a reduction in pain intensity as treatment progresses. During the initial alignment and leveling phase of fixed orthodontic treatment, orthodontists typically use nickel–titanium archwires known for generating light, continuous forces [13,14]. Interestingly, although pain lasting one week may seem uncommon, the data show that patients in their first six months of treatment reported it more frequently. This may reflect heightened sensitivity and anxiety during the initial adaptation period [15]. All patients in the fixed appliance group began treatment with 0.014-inch super-elastic nickel–titanium archwires, designed to exert light, continuous forces. Despite this, early-stage discomfort can be perceived as more intense due to psychological and emotional factors, especially in patients without previous orthodontic experience [15,16].

The obtained results align with other studies that reported that discomfort and pain related to orthodontic appliances usually subside in the range of 4–7 days, with fixed appliances inducing more severe discomfort than clear aligners [2]. Similarly, Cardoso et al. [17] noted that patients treated with clear aligners experienced significantly less pain than those treated with fixed appliances, particularly during the initial days of treatment. This can be attributed to the lower pressure, tension, and sensitivity experienced by clear aligners users [18]. Conversely, Chan et al. [19] found comparable pain levels in both groups two days post-insertion, although fixed appliance users experienced a pain peak two days after each adjustment, often persisting for up to seven days following the introduction of a new archwire [19,20].

It was also found that patients undergoing fixed orthodontic treatment experienced more frequent masticatory difficulties compared to those with clear aligners, likely due to the continuous forces exerted by fixed systems. Inauen et al. [21] reported that pain during chewing, biting, or intercuspation is more intense than spontaneous pain, as the already sensitized periodontal ligament is compressed by masticatory forces. Additionally, many orthodontists recommend a soft diet to minimize pain and prevent appliance breakage, which can influence patients’ nutritional status [22]. Minimizing appliance breakage can improve patient satisfaction and appointment efficiency [23].

Recent studies suggest that these dietary adaptations may have beneficial effects, as patients tend to avoid sticky foods, such as chocolates and snacks. Nonetheless, most continue to consume cereals, green leafy vegetables, fish, milk, and oils, maintaining a balanced diet consistent with orthodontic dietary recommendations [24,25].

Another noteworthy finding is that patients undergoing treatment for more than two years reported no weight loss, in contrast to those in earlier stages. This may be explained by a return to pre-treatment eating habits after an adaptation period of around three months. Similar findings were reported by other authors [26], who showed that orthodontic patients often experience weight loss during the first two months. However, others observed a return to pre-treatment BMI after three months, reinforcing the idea that most patients adapt to discomfort and dietary restrictions within that period [1].

In the studied sample, most patients (77.5%) were from urban areas, which may reflect greater accessibility to orthodontic services and increased awareness of alternative treatment options, such as clear aligners. Although residence was not used as a stratification variable in the statistical models, it is plausible that patients from urban environments had better access to information, pain management strategies, and newer orthodontic technologies [27]. In contrast, patients from rural areas may be more likely to receive fixed appliance treatment due to availability, cost considerations, or limited local orthodontic options [28,29]. These sociodemographic differences could influence not only treatment selection, but also the perception and reporting of pain, and should be further investigated in future research.

In this study, patients undergoing fixed appliance treatment also reported more frequent analgesic use compared to those with clear aligners. Those treated for less than six months typically reported discontinuous pain, whereas those treated for more than two years described their pain as continuous. This may be due to the uninterrupted force applied by fixed appliances, in contrast to clear aligners that patients can take off to relieve discomfort [3,5]. Although pain duration did not differ significantly between genders, female patients reported significantly higher analgesic use. This could suggest a more proactive approach to pain management among females, potentially contributing to equivalent or even lower perceived pain despite no observed differences in reported duration. However, our data do not directly analyze the impact of analgesic use on pain experience stratified by gender. Future studies should explore this relationship in more detail.

The lower pain levels reported with clear aligners may be explained by the dynamics of inflammatory mediators. Short-term inflammation induces sensitization, while prolonged exposure can lead to hyperalgesia [30]. Fixed appliances can trigger high discomfort following adjustment due to compression of the periodontal ligament, which activates nociceptors sensitized by inflammatory mediators. These mediators peak within 24 h and usually return to baseline within 7 days [31]. Therefore, the intermittent forces associated with clear aligners may lead to reduced nociceptive activation and pain [32].

Additionally, analgesic use may affect orthodontic treatment duration, as certain drugs have been shown to reduce tooth movement rates in animal models [33,34]. However, other studies suggest that relatively few patients use analgesics for pain relief in clinical settings [35,36].

The limitations of this study include the lack of a control group, which hinders causal interpretation, and the relatively small number of patients treated with clear aligners. Additionally, pain perception was self-reported and therefore subjective. We also lacked baseline and follow-up weight data, which prevented a detailed analysis of weight progression across different patient profiles (e.g., overweight vs. normal weight). Another limitation of this study is the absence of systematic data regarding the use of intermaxillary elastics. Although elastics were used in some treatment plans, they were not included in the questionnaire and thus not analyzed. Given their potential influence on pain perception and dietary adaptations, future research should consider incorporating auxiliary mechanics into study design and data collection.

Furthermore, the presence of a researcher during questionnaire completion may have introduced observer bias, potentially influencing participants’ responses. Finally, the absence of standardized pain assessment tools, such as the visual analog scale (VAS), limits the objectivity and comparability of our reported pain measures, warranting the inclusion of validated pain assessment instruments in the subsequent research.

Future research should focus on longitudinal monitoring of individual changes in pain perception, dietary behavior, and weight trends throughout orthodontic treatment, considering variables such as malocclusion severity, treatment modality, and the consistent use of auxiliary mechanics, such as intermaxillary elastics. Given the potential for observer bias identified in this study, future designs should prioritize anonymous or independently administered data collection methods to enhance the validity of patient-reported outcomes. Additionally, future research should preferably utilize standardized and validated tools, like the visual analog scale (VAS), to objectively quantify pain experiences. Incorporating assessments of socioeconomic status and geographic factors would also enhance understanding of disparities in orthodontic care. Ultimately, addressing these factors comprehensively could significantly improve pain management strategies, patient counseling protocols, and overall quality of care, fostering stronger orthodontist–patient relationships and greater treatment adherence.

## 5. Conclusions

This study assessed how orthodontic appliance type and treatment duration influence pain perception, analgesic use, food impairment, and weight loss. Patients with fixed appliances reported longer pain latency, greater analgesic use, and more frequent dietary limitations compared to those with clear aligners. These findings highlight distinct discomfort profiles between treatment modalities and may help clinicians improve patient counseling and pain management strategies to enhance adherence and treatment outcomes.

## Figures and Tables

**Table 1:** Orthodontic pain and dietary impact questionnaire.

Item No.	Question	Answer Options
1	What is your age?	(a) 6–12 years (b) 13–18 years (c) Over 18 years
2	What is your gender?	(a) Female (b) Male
3	What is your residential environment?	(a) Urban (b) Rural
4	What is your educational status?	(a) Pupil (b) Student (c) Employee
5	What type of orthodontic treatment are you undergoing?	(a) Clear aligner (b) Fixed appliance
6	How long have you been wearing your orthodontic appliance?	(a) Less than 6 months (b) 6 months–1 year (c) 1–2 years (d) More than 2 years
7	How long did the pain last after the most recent adjustment or tray change?	(a) Several days (b) One week
8	How long after the adjustment/tray change did the pain begin?	(a) No pain (b) 5 min (c) Several hours (d) One day (e) One week
9	Was the pain continuous or intermittent?	(a) Continuous (b) Intermittent
10	Has your ability to eat been affected by the orthodontic treatment?	(a) Yes (b) No
11	Have you changed the types of food you eat since starting orthodontic treatment?	(a) Yes (b) No
12	Have you noticed any weight loss since starting orthodontic treatment?	(a) Yes (b) No
13	Have you ever taken painkillers after an adjustment or tray change?	(a) Yes (b) No

**Table 2:** Demographic and clinical characteristics of the study population based on questionnaire responses.

Category	Number (%)
**Age**	
6–12 years	26 (16.3%)
13–18 years	82 (51.2%)
>18 years	52 (32.5%)
**Gender**	
Female	120 (75.0%)
Male	40 (25.0%)
**Environment**	
Rural	36 (22.5%)
Urban	124 (77.5%)
**Educational status**	
Pupil	114 (71.2%)
Student	20 (12.5%)
Employee	26 (16.3%)
**Type of orthodontic treatment**	
Fixed	134 (83.7%)
Clear aligners	26 (16.3%)
**Duration of orthodontic treatment**	
<6 months	32 (20.0%)
6 months–1 year	52 (32.5%)
1 year–2 years	64 (40.0%)
>2 years	12 (7.5%)
**Pain duration**	
Several days	146 (91.3%)
One week	14 (8.8%)
**Pain latency**	
No pain	14 (8.8%)
5 min	34 (21.3%)
Several hours	64 (40.0%)
One day	46 (28.7%)
One week	2 (1.3%)
**Pain continuity**	
Discontinuous	128 (80.0%)
Continuous	32 (20.0%)
**Food impairment**	
Present	70 (43.8%)
Absent	90 (56.2%)
**Diet change**	
Present	76 (47.5%)
Absent	84 (52.5%)
**Weight loss**	
Present	48 (30.0%)
Absent	112 (70.0%)
**Use of analgesics**	
Present	84 (52.5%)
Absent	76 (47.5%)

**Table 3:** Association between pain duration and patient age and gender.

Variable	Category	Several Days (*n*, %)	One Week (*n*, %)	*p*-Value
**Age**	≤18 years	122 (91.0%)	12 (9.0%)	0.780 *
	>18 years	56 (93.3%)	4 (6.7%)	
**Gender**	Female	94 (90.4%)	10 (9.6%)	0.603 *
	Male	84 (93.3%)	6 (6.7%)	

* Fisher’s exact test.

**Table 4:** Association between analgesic use and patient gender and residential environment.

Variable	Category	Absent (*n*, %)	Present (*n*, %)	*p*-Value
**Gender**	Female	38 (36.5%)	66 (63.5%)	0.021 *
	Male	48 (53.3%)	42 (46.7%)	
**Environment**	Rural	20 (34.5%)	38 (65.5%)	0.083 *
	Urban	66 (48.5%)	70 (51.5%)	

* Fisher’s exact test.

**Table 5:** Association between appliance type (fixed vs. clear aligners) and pain latency, food impairment, and use of analgesics.

Variable	Category	Fixed Appliances (*n*, %)	Clear Aligners (*n*, %)	*p*-Value	Odds Ratio (95% CI)
**Pain** **Latency**	No pain	10 (71.4%)	4 (28.6%)	<0.001 *	0.079 (0.029–0.216)
	5 min	18 (52.9%)	16 (47.1%)		
	Several hours	58 (90.6%)	6 (9.4%)		
	One day	46 (100.0%)	0 (0.0%)		
	One week	2 (100.0%)	0 (0.0%)		
**Food** **Impairment**	Absent	70 (77.8%)	20 (22.2%)	0.020 *	3.048 (1.152–8.066)
	Present	64 (91.4%)	6 (8.6%)		
**Analgesics** **Use**	Absent	54 (71.1%)	22 (28.9%)	<0.001 *	8.148 (2.659–24.970)
	Present	80 (95.2%)	4 (4.8%)		

* Fisher’s exact test.

## Data Availability

The data presented in this study are available on request from the corresponding authors. The data are not publicly available due to privacy reasons.
